# Quantitative Analyses of Force-Induced Amyloid Formation in *Candida albicans* Als5p: Activation by Standard Laboratory Procedures

**DOI:** 10.1371/journal.pone.0129152

**Published:** 2015-06-05

**Authors:** Cho X. J. Chan, Ivor G. Joseph, Andy Huang, Desmond N. Jackson, Peter N. Lipke

**Affiliations:** 1 Biology Department, Brooklyn College City University of New York, New York, New York, United States of America; 2 The Graduate Center, City University of New York, New York, New York, United States of America; 3 Haskins Laboratories and the Department of Chemistry and Physical Sciences, Pace University, New York, New York, United States of America; Universitat Autònoma de Barcelona, SPAIN

## Abstract

*Candida albicans* adhesins have amyloid-forming sequences. In Als5p, these amyloid sequences cluster cell surface adhesins to create high avidity surface adhesion nanodomains. Such nanodomains form after force is applied to the cell surface by atomic force microscopy or laminar flow. Here we report centrifuging and resuspending *S*. *cerevisiae* cells expressing Als5p led to 1.7-fold increase in initial rate of adhesion to ligand coated beads. Furthermore, mechanical stress from vortex-mixing of Als5p cells or *C*. *albicans* cells also induced additional formation of amyloid nanodomains and consequent activation of adhesion. Vortex-mixing for 60 seconds increased the initial rate of adhesion 1.6-fold. The effects of vortex-mixing were replicated in heat-killed cells as well. Activation was accompanied by increases in thioflavin T cell surface fluorescence measured by flow cytometry or by confocal microscopy. There was no adhesion activation in cells expressing amyloid-impaired Als5p^V326N^ or in cells incubated with inhibitory concentrations of anti-amyloid dyes. Together these results demonstrated the activation of cell surface amyloid nanodomains in yeast expressing Als adhesins, and further delineate the forces that can activate adhesion *in vivo*. Consequently there is quantitative support for the hypothesis that amyloid forming adhesins act as both force sensors and effectors.

## Introduction

Yeast cell surface adhesins, such as the *Candida albicans* adhesin Als5p and *Saccharomyces cerevisiae* flocculins Flo1p and Flo11p, mediate cell-to-cell aggregation and cell-to-surface adhesion. Within the mid-regions of many adhesins are 6-7-amino acid sequences predicted by TANGO (http://tango.crg.es/) to form amyloids [1–3]. A single site mutation (V326N) in the amyloid region of Als5p decreases cell-to-cell aggregation, cell-to-substrate adhesion, and fluorescence of the amyloid-reporting dye thioflavin T [4]. Similarly, anti-amyloid compounds inhibit activation of the *S*. *cerevisiae* flocculins [2].

Extension forces cluster the adhesins into amyloid-like surface patches [4,5]. Mechanical extension force applied with the tip of an atomic force microscope (AFM) activates the clustering of Als5p (hereafter designated Als5p^WT^) molecules into nanodomains and the clusters propagate across the cell surface. This clustering is mediated by the amyloid-forming sequence, because the clustering response is absent from a non-amyloid-forming mutant of the protein Als5p^V326N^ [2,4,5]. We have proposed that the pulling on the surface protein results in exposure of the amyloid regions of the protein, which then interact through amyloid stacking to cluster with neighboring Als5p^WT^ molecules in 100–500nm diameter surface nanodomains [4,6]. These nanodomains are highly fluorescent after staining with thioflavin dyes [4,5]. Als5p^WT^ clusters take minutes to form and propagate slowly around the cell surface at a rate of ~20 nm/min. Similarly, hydrodynamic shear from laminar flow can also activate the yeast surface amyloids to increase surface binding, cell-cell aggregation, and formation of mechanically robust biofilms [7]. These changes are consistent with observations that *C*. *albicans* biofilms grown under flow are more extensive and include more hyphae [8].

These findings correspond to known properties of amyloids. One relevant observation is that shear force can partially unfold proteins, leading to exposure of amyloid-forming sequences. Subsequently these sequences aggregate into β-sheet rich forms that assemble in a cross-β structure, characteristic of amyloid fibril formation [9–14]. For instance, conformational changes in proteins resulting from partial unfolding from their native state facilitate amyloid formation in transthyretin [15] and lysozyme [16]. Shear flow from a Couette cell produces amylogenic precursors in β-lactoglobulin, and enhances fibril formation as well through the alignment and further unfolding of the protein under shear flow, thus resulting in the formation of amyloid precursors and or their maturation into fibers [17,18].

Secondly, amyloid formation itself may be triggered by shear force. When Aβ-peptide is stirred there is an increase in thioflavin T fluorescence as well as growth of amyloid fibers that are not seen with quiescent peptides [18]. Dunstan et al. hypothesized that a possible mechanism of the effect of shear is the alignment of the aggregates, to facilitate assembly into fibrils. This idea is supported by observations that aggregates of proteins such as β-lactoglobulin align under flow [17,19].

Testing such ideas in the yeast adhesins requires the ability to quantify amyloid formation, something we have not been able to do *in vivo*. Therefore we set out to induce and measure activation of adhesion in populations of cells. The development of these assays has led to our realization that the cell surface adhesins are sensitive to activation during cell preparation. Quantitative assays also have confirmed that amyloid-forming adhesins can both sense and respond to force.

## Results

### Effect of vortex-mixing on adhesion of Als5p-expressing *S*. *cerevisiae* cells

**Fig 1:**
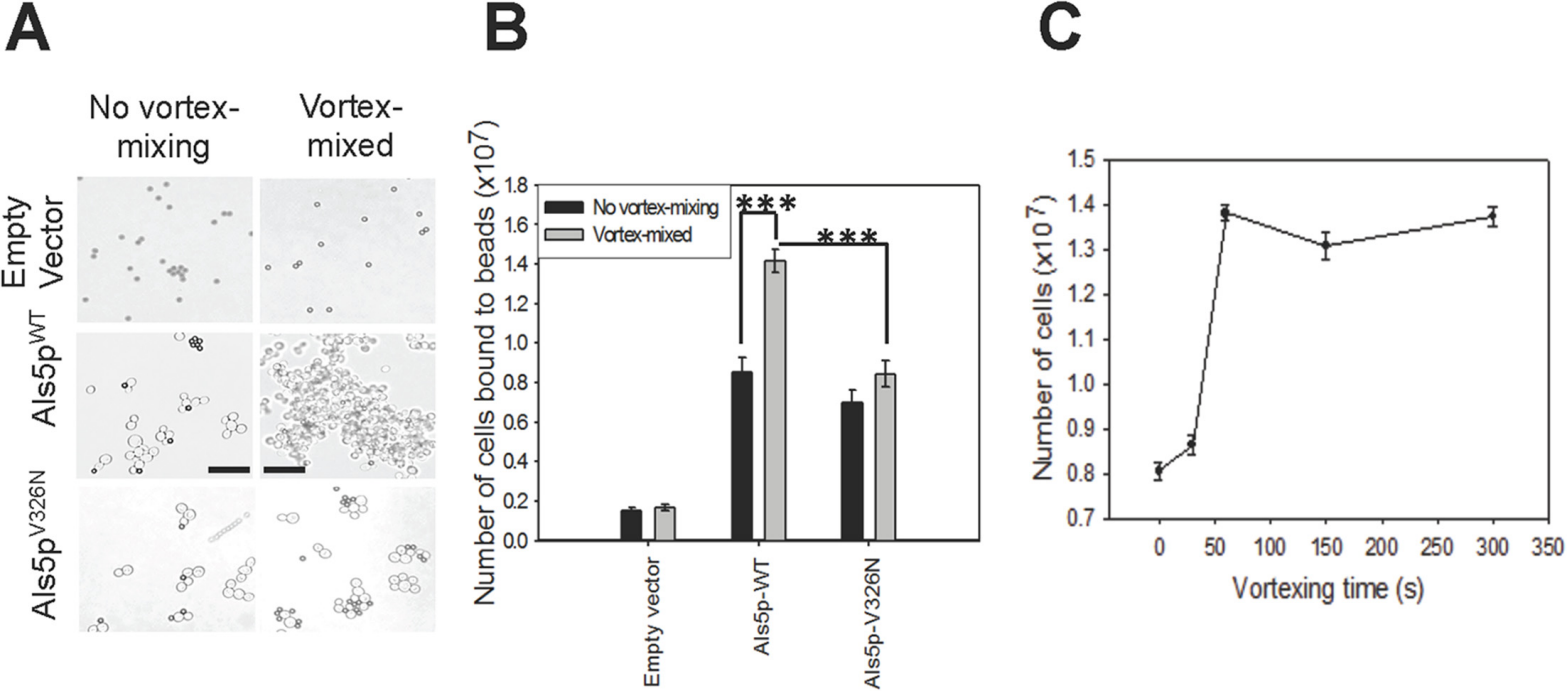
The effects of vortex-mixing on adhesion and aggregation of *S*. *cerevisiae*. Cells carrying an empty vector or expressing Als5p^WT^ or Als5p^V326N^ were vortex-mixed or not, then aggregated for 10 minutes with heat-denatured BSA-coated magnetic beads: **(A)** Bright-field micrographs of the cells. The dark-colored beads are 1 μm in diameter. Scale bars represent 20μm. **(B)** Quantification of cells adhering to beads. Error bars represent s.d. for n = 4. A student t-test was performed: *** represents p<0.001. **(C)** Time course for activation of Als5p-expressing cells by vortex mixing at 2500 rpm. Error bars represent s.e.m. for n = 8.

We looked for increases in cell-to-bead adhesion and cell-to-cell aggregation of Als5p^WT^-expressing cells with ligand-coated beads [20]. Suspensions of cells expressing Als5p^WT^ were vortex-mixed for 5 minutes at 2500 rpm. The initial onset of adhesion was determined by monitoring size of aggregates in the first 10–15 minutes of aggregation. (These brief assays minimized induction of nanodomains that occurs during standard 45 min assays [4,20]). Cells that had been vortex-mixed formed bigger initial aggregates than cells that were not vortex-mixed (Fig 1A). To quantify the number of cells bound we suspended the aggregates with NaOH and then determined optical density at 600nm. Vortex-mixing of the cells caused an average 1.6-fold increase in adhesion to beads and aggregation (Fig 1B). There was no aggregation in cells expressing empty vector when vortex-mixed (Fig 1A and 1B).

We determined the vortex-mixing time needed to initiate cell adhesion. Mixing for 60 seconds increased the number of cells bound to the beads from (8.1 ±. 4) x 10^6^ to (1.4 ±. 03) x 10^7^. There was no additional increase with mixing times up to 5 min (Fig 1C). Therefore under these conditions 60 seconds of vortex-mixing was sufficient to increase the adhesion and aggregation of the cells to ligand-coated beads.

### Amyloid-dependence of vortexed-induced adhesion

**Fig 2:**
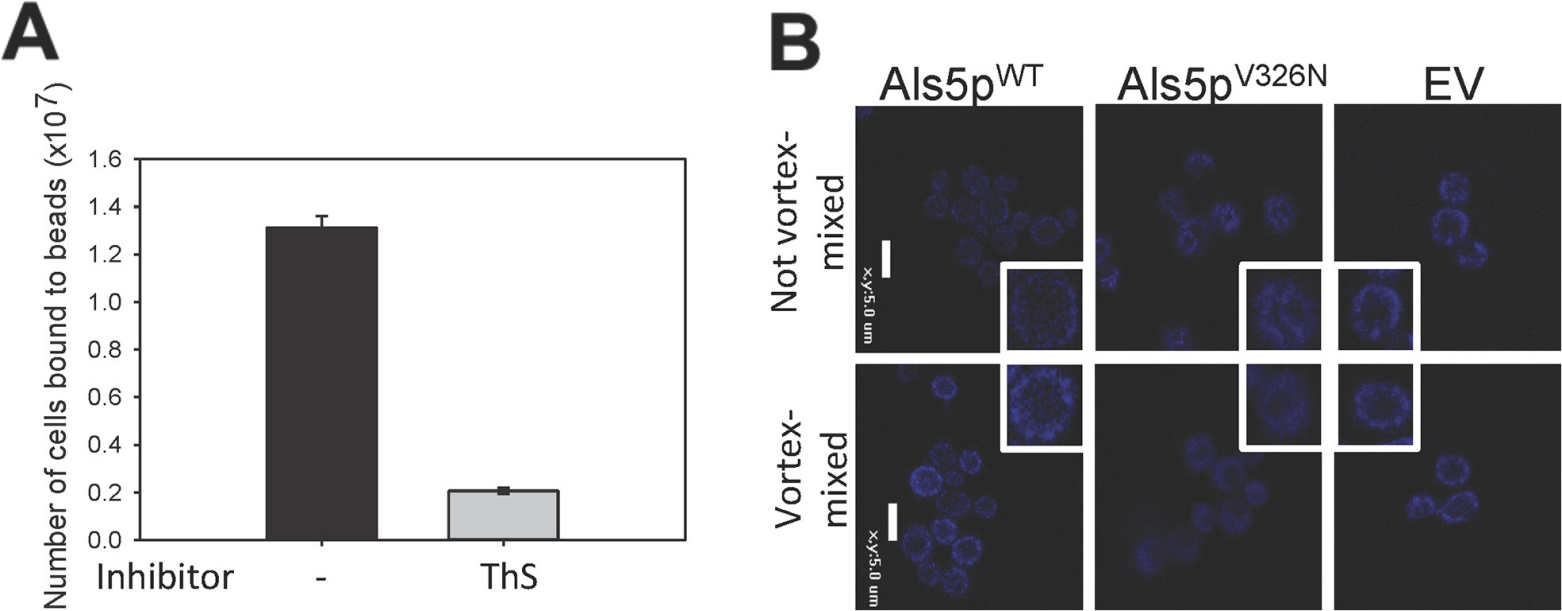
(A) Effects of anti-amyloid dye on vortex-activated adhesion and aggregation of *S*. *cerevisiae* cells expressing Als5p^WT^. A 10- minute aggregation assay in the absence or presence of 0.2 mM ThS. **(B)** Effects of vortex mixing on surface amyloid nanodomains. Confocal micrographs of cells stained with 500 nM ThT without vortex mixing (top row) or after 5 min vortex mixing (bottom row). Scale bars represent 5μm.

If force-induced cell adhesion is amyloid-dependent, then vortex-mixing should not activate aggregation on cell expressing the non-amyloid mutant Als5p^V326N^ adhesin. As predicted, there was no increase in the size of the aggregates of the amyloid mutant protein (Fig 1A and 1B). Similarly, amyloid-binding dyes should inhibit this increase. At concentrations above 30 μM, the amyloid-binding dye thioflavin S (ThS) binds to and disrupts amyloids, therefore decreasing adhesion [2,4,21]. This was indeed the case: ThS (0.2 mM) added after vortex-mixing inhibited the binding of Als5p^WT^-expressing cells to the ligand-coated beads by 6.3-fold (Fig 2A). In the presence of ThS, there were no aggregates formed with Als5p^V326N^-expressing cells or cells with empty vector, nor was there any effect of vortex-mixing (data not shown).

### Effect of vortex-mixing on cell surface thioflavin-T fluorescence

Amyloid-dependent activation of cell adhesion is mediated by formation of surface amyloid nanodomains that migrate around the cell surface [5]. Sub-inhibitory concentrations of thioflavin T (ThT) or ThS stain amyloids *in vitro* and on yeast cell surfaces [2,4,9,17]. To assay whether the increase in adhesion was accompanied by the formation of amyloids on the surface of cells expressing yeast adhesins, we stained quiescent and vortex-mixed cells with ThT or ThS (1 μM, a non-inhibitory concentration) and analyzed them by flow cytometry. For Als5p^WT^-expressing cells, vortex-mixing increased the surface fluorescence (S1 and S2 Figs). This increase in surface fluorescence was not seen with cells transformed with EV or cells expressing the non-amyloid Als5p^V326N^.

The mean cellular fluorescence from flow cytometry correlated with data that vortex-mixing increases the mean cell surface fluorescence (Table 1 and S1 Table). There was a 1.5-fold fluorescence increase due to vortex mixing for cells expressing Als5p^WT^. Cells with EV or expressing Als5p^V326N^ had little to no increase. Results were similar with ThS (S2 Fig and S1 Table). Therefore, vortex-mixing cells expressing Als5p led to significant increases in surface fluorescence intensity with the amyloid-staining dyes.

**Table 1:** Effect of vortex-mixing on mean ThT fluorescence of yeast cells.

Yeast	Mean ± se		Ratio
	Vortex-mixed	Quiescent	
*C*. *albicans*	792 ± 57	541 ± 61	1.51 ± 0.12
*S*. *cerevisiae* (Als5p^WT^)	317 ± 25	207 ± 18	1.53 ±. 0.12
*S*. *cerevisiae* (Als5p^V326N^)	85 ± 37	99 ± 48	0.85 ± 0.66
*S*. *cerevisiae* (EV)	72 ± 24	70 ± 13	1.03 ± 0.38

Nanodomains formed by vortex mixing were also microscopically visible. Cells were vortex-mixed for 5 min. and then stained with ThT (Fig 2B). This increase was not seen with the non-amyloid mutant Als5p^V326N^ or cells with empty vector. These data confirmed that vortex-mixing induced formation of ThT-fluorescent surface nanodomains in cells expressing a yeast adhesin.

### Effects of vortex-mixing in *C*. *albicans*

**Fig 3:**
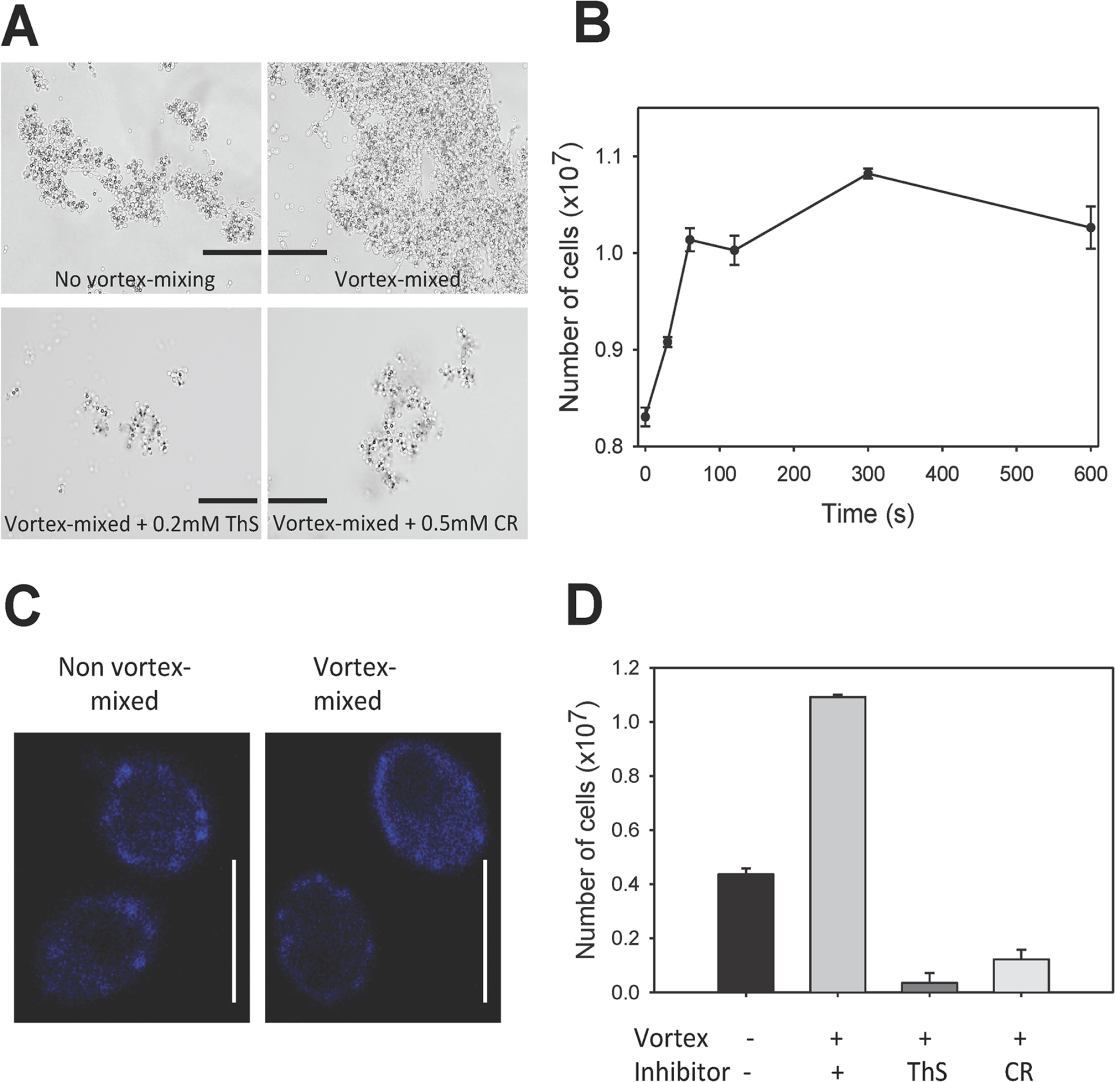
Effect of vortex-mixing on aggregation and surface nanodomains on *C*. *albicans* SC5314 cells. **(A)** Effects of vortex mixing on initial aggregation. Scale bars represent 100μm. **(B)** Number of cells bound to beads after vortex mixing for different times. Error bars represent standard deviation for n = 3. (**C)** ThT staining of control and vortex-mixed SC5314 cells. Scale bars represent 5μm. **(D)** Inhibition of aggregation with amyloid dyes 0.2mM ThS and 0.5mM Congo red after vortex-mixing.

We also assayed increases of aggregation in live *C*. *albicans* cells. To maximize expression of Als1p and perhaps other adhesins, cells were diluted in fresh YPD media for 45 minutes before the assays [22,23]. When *C*. *albicans* cells were vortex-mixed at 2500 RPM for 5 min., the initial aggregates were larger than in cells not vortex-mixed (Fig 3A). The mixing time for maximal activation was similar to that for Als5p^WT^-expressing *S*. *cerevisiae*, but the initial rate of activation was greater, with a 10% increase within 30 seconds of vortex-mixing and a maximal 2.75-fold increase in initial adhesion rate (Fig 3B and 3D). Activation was accompanied by a slight increase in surface ThT fluorescence (S1 and S2 Figs, Table 1, and S1 Table). Confocal microscopy showed that vortex-mixing induced subtle differences in *C*. *albicans* surface structure, with some cells having a more uniform and less punctate distribution of surface amyloid (Fig 3C). As with Als5p^WT^, ThS (200 μM) or CR (500 μM) inhibited the aggregation and adhesion of cells to ligand-coated beads (Fig 3D).

### Effects of vortex-mixing of heat-killed adhesin-expressing cells

Heat-killed cells aggregate effectively, and are able to form surface nanodomains [4,5]. Therefore, if vortex-mixing-induced nanodomain formation is independent of cellular metabolism, it should also be apparent in heat-killed cells. Cells expressing Als5p^WT^ or Als5p^V326N^ were heat-killed for 15 minutes at 60°C, then allowed to equilibrate at 25°C for one hour before assay. As expected, there was an increase in bright puncta on the surface of heat-killed Als5p^WT^ cells when vortex-mixed (S3 Fig). In contrast, heat-killed Als5p^V326N^ non-amyloid mutant cells did not show annular staining or nanodomain formation.

### Effects of centrifuging cells on aggregation

**Fig 4:**
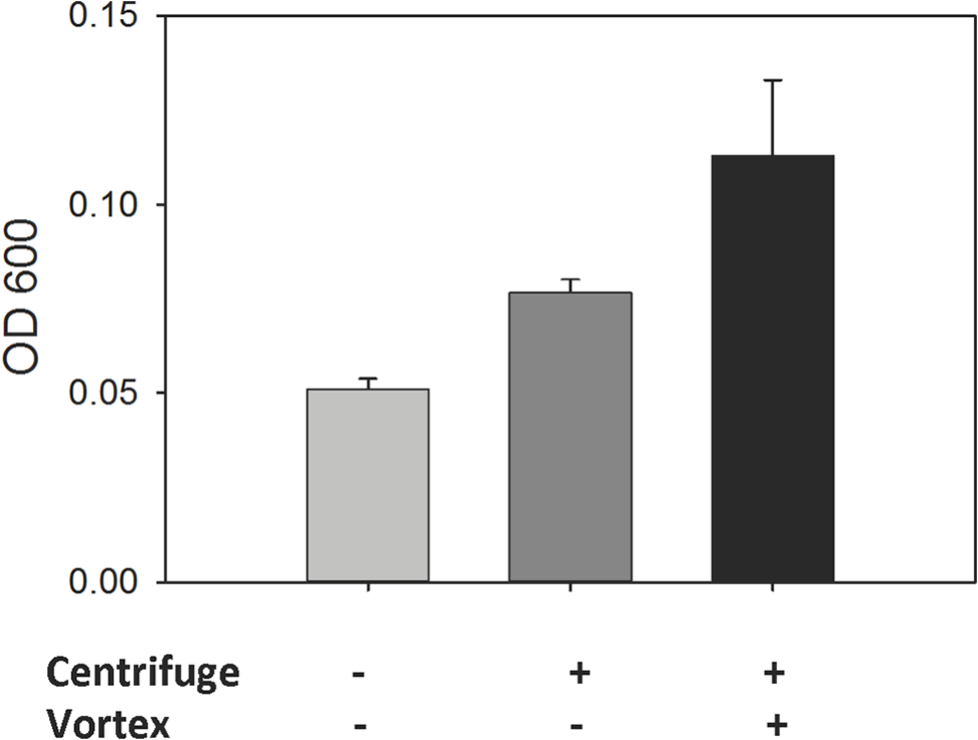
Effect of centrifugation on aggregation. Cells were grown in buffered medium, and aggregated in 10 min. assays. Some of the cells were centrifuged (3200 x g, 3mins) and resuspended before assay, and some of the centrifuged cells were also vortex mixed (2500 rpm, 1 min.) before assay.

Because vortex mixing activated amyloid-dependent cell adhesion, it was possible that there might be similar effects from the shear associated with centrifugation and resuspension of the cells. Therefore we grew cultures in medium buffered at pH 5.5 with 50mM MOPS so that aggregation assays could be performed directly on cells. This procedure eliminated the need to centrifuge the cells and resuspend in buffer before assay. Culture aliquots of 1ml were placed directly in test tubes. The samples were then treated in one of three procedures: some tubes were left quiescent, some were centrifuged and the cells resuspended, and some were centrifuged, resuspended, and then vortex-mixed at 2500 rpm for 1 min. Aggregation assays determined the initial rates (Fig 4). Centrifugation increased the initial aggregation values 1.7-fold. Vortex-mixing activated the cells another 1.6-fold. The total fold increase due to centrifugation, resuspension, and vortex mixing was 2.7-fold. Therefore, standard techniques for washing and resuspension of cells can activate formation of amyloid surface nanodomains.

## Discussion

Our study of activation of aggregation in cell suspensions reinforces the idea that force alone is sufficient to induce surface nanodomains [4,7,24]. This remarkable activity takes place on cell surfaces without need for a signaling or metabolic response in the cell [4,5]. The results also show that shear forces for formation of amyloid nanodomains are similar to those applied to cells in standard laboratory protocols for washing and resuspending yeasts, and vortex mixing. Therefore cell preparation procedures can inadvertently affect aggregation behavior. We have also demonstrated quantification of amyloid formation by flow cytometry and quantification of initial rates of cell adhesion. The techniques and results are valid for *C*. *albicans* as well as for Als5p displayed on the surface of *S*. *cerevisiae*.

### Fungal adhesins as force sensors

Our results constitute additional support for the idea that Als adhesins themselves sense and respond to force [5,7]. There is a simple model that explains this idea. First, the amyloid sequence-containing T domain in Als5p^WT^ is only marginally stable, and the domain unfolds in response to extension forces [5,7]. Domain unfolding exposes the amyloid core sequence, which in turn interacts with amyloid sequences in nearby Als adhesin molecules to form cell surface nanodomains within minutes [4–6,24–26]. These nanodomains consist of arrayed adhesin molecules, aggregated on the cell surface through amyloid-like interactions of the amino acids in amyloid core sequences [27,28]. Thus, amyloid formation is a consequence of protein conformational change, and depends on presence of a functional amyloid-forming sequence in the T domain of Als proteins [4,5]. The clustering results in very high local concentrations of adhesins, and consequent reduction in macroscopic *k*_*off*_ values [6].

This activation is cell-autonomous in the sense that the force sensors and the responding effectors are the same molecules, namely the adhesin molecules already present at the cell surface [5,7]. Expression of adhesin Als5p on the surface of *S*. *cerevisiae* is sufficient to allow amyloid nanodomain formation and activation of cell aggregation in response to force (Fig 1) [4,29].

This response to force is a general mechanism, rather than a response to a specific kind of force. The AFM experiments show that extension force in one area of the cell can lead to activation of the entire cell as the nanodomains propagate across the surface [6,7]. Similarly, a single area of the cell surface is initially stimulated when a cell binds to a ligand-coated bead; mixing during the adhesion assay generates extension force on the adhesins bound to the bead [21]. Later in the assay, cell-to-cell adhesion may directly stimulate adhesins on parts of cells that are not in contact with a bead. Activation can also follow more global stimulation, as in vortex mixing or laminar flow [7]. Vortex-mixing suspensions at different cell densities did not show differences in activation rates, and this result implied that liquid shear was the activating force, rather than cell-cell collisions. Als protein surface amyloids are present on *C*. *albicans* abscesses in infected tissue, so the nanodomains must form during the infection process, perhaps due to friction exerted by fungal growth through the host tissue [30,31]. Thus, the data strengthens a generally applicable model of force-activated fungal cell adhesion [4,7,24].

### Time and force for activation

Nanodomain formation followed after one minute of vortex mixing. This time is significantly shorter than the 25 min interval observed after single molecule stimulation for Als5p^WT^ in AFM experiments [5]. On the other hand, Als-bearing cells start to become globally cell surface activated within 15 minutes in adhesion assays, and 7 minutes under laminar flow [7]. These differences in activation rate are consistent with differences in the frequency of molecular stretching in the three different scenarios. A few molecules are individually stretched in the AFM. In our adhesion assays, cells are gently mixed, usually at 170–200 rpm. This process results in random collisions, adhesions and subsequent stretching of adhesins, so many areas of the cell surface are stimulated in the course of a few minutes. Our unpublished data shows that slower vortex mixing speeds are less effective, and that mixing speed in the adhesion assay itself affects the size of the aggregates, with greater adhesion as the mixing speed becomes faster. Therefore, the speed of mixing both before and during the adhesion assay affect initial rate of cell-to-cell aggregation.

The forces needed to activate cell adhesion are comparable to those the yeast encounter *in vivo* and in the lab. Unfolding of the amyloid-containing T domains of Als5p^WT^ or Als1p in the AFM followed application of forces in the range of 50–100 pN [5,26,29]. This amount of force is similar to that encountered in flowing blood, or under flow in the natural environment [32]. Physiological shear rates *in vivo* are in the range of 100–8000 s^-1^ in blood vessels and the extracellular matrix [32]. Such a shear rate is also similar to that applied by vortex mixing, where a broad range of shear rates, from 200–8000 sec^-^1, occur depending on sample volume, proximity to air and glass interfaces, vessel geometry, and mixing speeds [33]. The product of shear rate and viscosity (8.9 x 10^–3^ dyne sec cm^-2^) yields a resulting shear stress of ~10–100 dyne cm^-2^, or 1–10 pN μm^-2^. Although this force appears less than the instantaneous force applied in AFM, its application over one minute time would lead to high T domain unfolding probability, relative to the standard AFM contact time of a second or less [29]. Thus, Als proteins show activation under forces such as centrifugation or vortex mixing, as we have demonstrated. Therefore, cell preparation procedures will affect results of cell adhesion assays, and cells will need to be treated gently to achieve baseline aggregation ability (the equivalent of the quiescent cells in our assays).

The consequences of force-induced activation can be easily quantified. Confocal microscopy and flow cytometry with thioflavin T are effective in visualizing and quantifying surface amyloids. Force-activated cells had punctate nanodomains with increased thioflavin T staining, which may also be measured by image analysis (not shown). Collectively, the assays can now be used to quantify amyloids in other fungal adhesins. We have also shown that quantities of adhering cells can be compared by spectrophotometry instead of cell counting after cell dissociation [20]. This procedure has allowed us to measure initial rates of adhesion.

### Summary

We have outlined quantitative methods for comparison of cell surface amyloid nanodomains and for cell adhesion in a magnetic bead assay, including showing differences in initial adhesion rate for vortex-stimulated cells. We have also shown that vortex-mixing measurably and reproducibly activated surface amyloid nanodomain formation on populations of cells, allowing us to compare assays on different days and cell cultures. The results support a conclusion that the mechanism and consequences of activation by laminar flow, vortex-mixing, or shaking in aggregations assays are similar. Each of these techniques shows increased surface fluorescence with thioflavin T, sensitivity to anti-amyloid dyes, and comparable kinetics and force requirements. Bioinformatic analyses show that similar amyloid-forming sequences are common in fungal adhesins, as well as some bacterial adhesins [34–37]. Indeed the importance of amyloid interactions has also been demonstrated in several other fungal and bacterial adhesion systems, including assembly of gram negative curlins [38–41], and gram positive adhesins including *Streptococcus mutans* P1 [42], and *Bacillus subtilis* TasA [36,43]. It remains to be seen if any of these other systems also show force-induced clustering and activation.

## Materials and Methods

### Strains and media

*Saccharomyces cerevisiae* strain W303-1B *MATα leu2 ura3 ade2 trp1* (Rodney Rothstein, Columbia U.) harboring the empty vector (pJL1-EV) or expressing Als5p^WT^ or Als5p^V326N^ was grown in complete synthetic medium (CSM) lacking tryptophan with galactose as carbon source [4]. Cultures were grown for 48 hours at 24°C at 170 RPM. When desired, cells were heat killed in a water bath at 60°C for 15 minutes and then incubated at room temperature for 1 hour before activation and assay.

*Candida albicans* strain SC5314 was grown overnight in yeast extract with peptone and 2% glucose (YPD) at 30°C at 170 RPM. Als1p expression was induced by placing an aliquot of cells in fresh YPD medium [23,44] in a 1:10 dilution and shaking at 170 RPM at 30°C for 45 minutes.

### Aggregation assays

Aggregation assays were modified from published procedures [20,21]. Briefly, cells were centrifuged at 4000 RPM for 3 minutes to remove culture media. The cells were then washed gently three times with 10mM tris, 1mM EDTA (TE) pH 7.0 and gently resuspended in the same buffer. The OD_600nm_ of the cell suspension was determined with a Spectronic 21 D+ spectrophotometer, and the suspension was adjusted to 10^8^ cells/ml. Aliquots (1 ml) were then placed in test tubes (13x100 mm) either left stationary on the lab bench or vortex-mixed at 2500 RPM for 5 min using a Fisher Scientific multi-tube vortexer. This vortex mixer has an eccentric orbit of 3.6mm. (Standard lab vortex mixers have 5 mm orbits.) Cell suspension (1ml) was mixed with 10^6^ BSA-coated magnetic beads. The suspensions were incubated on an orbital shaker for 10 minutes at 170 RPM at 24°C. The assay tubes were placed on a magnet. The unbound cells were gently removed with a pipette and the beads with the cell aggregates were washed once 500 μl of TE buffer. For microscopic viewing, cells were resuspended in 100 μl of TE buffer and 4 μl applied to a glass slide. Microscopic observations were made with an Olympus microscope using a 60X oil objective. For quantification, the aggregates and beads were resuspended in 300 μl 1 M NaOH and shaken gently on an orbital shaker for 20 minutes. The beads were then separated on a magnet, and the OD_600nm_ determined on a 200μl aliquot in a 96-well plate with a Spectronic Genesys plate reader. In this assay OD_600nm_ of 1.0 corresponds to 8.8 x 10^7^ cells/ml. Unless otherwise stated, all assays were done on at least two independent cultures, in triplicate for each.

M-280-tosylactivated-magnetic Dynabeads (Invitrogen, Carlsbad, CA) were covalently derivatized with 1mg/ml heat-denatured bovine serum album (BSA) overnight according to the manufacturer’s protocol.

### Dye inhibition

Als-expressing *S*. *cerevisiae* cells or *C*. *albicans* cells were vortex-mixed or left quiescent for 5 minutes and then ThS or CR) was added. Ligand-coated beads were added, and aggregation assays performed as described above.

### Staining protocols

Stock concentrations of ThS and ThT were made with deionized water and filtered with a 2 μm filter. The concentration was then determined with a spectrophotometer, using Beer’s law, using an extinction coefficient of 2.66 x 10^3^ L/mol*cm.

### Confocal microscopy

Confocal imaging was done with a Nikon confocal microscope. 10^8^ cells were stained with ThT (1 μM) in a final volume of 1ml immediately after vortex-mixing. The cells were vortex-mixed on a low setting with the dye for 5 seconds to resuspend the dye, and then 4 μl of the suspension was placed onto a glass slide for imaging. The stained cells were not washed prior to microscopy. The gain of the microscope was set at 7.75 with the phase at 162. The excitation was at 408nm with an emission detector at 450 ± 35 nm. Pictures were taken at 2048 x 2048 quality. The images were quantified for blue pixel counts using the Image J software with Color Profiler plugin. Six cells per sample were counted together for blue pixels.

### Flow Cytometry

Flow cytometry was done with BD Biosciences BD FACS Aria II cell sorter with excitation at 405 nm and an emissions filter of 450 ± 50nm. 10^6^ cells were in 12mm x 75 mm tubes with or without vortex-mixing and then brought to a final concentration of 1 μM ThT or 1 μM ThS in a total volume of 1 ml in their respective buffer as mentioned above. The cells were filtered with a 40 μm filter before analysis. A 70-micron nozzle size was used with default sheath pressure, amplitude, and frequency parameters as per manual. 20,000 cells were monitored for each assay.

### Centrifugation Aggregation Assay

Cells were grown in Complete Synthetic medium without Trp (Sunrise Science Products), with 40 mg/ml adenine, 2% galactose and 50mM MOPS, pH 5.5, over two nights to an OD 1.0 or more. Aliquots of 1 ml were gently pipetted into test tubes. Some tubes were left quiescent on the bench top, whereas others were centrifuged at 3200 x g for 3 min then gently resuspended in the same medium. Some tubes containing the centrifuged cells were then vortex-mixed at 2500 rpm for 1 minute. Ligand-coated magnetic beads were added to each tube at a 1:10 bead to cell ratio. 10 min aggregation assays were then performed.

## Supporting Information

S1 Fig Effects of vortex-mixing on ThT cell surface fluorescence. FACS analyses of populations of cells stained with ThT 1 μM. Yellow represents unstimulated cells, and blue represents vortex-mixed cells. **(A)** *S*. *cerevisiae* cells with EV; **(B)** *S*. *cerevisiae* cells expressing Als5p^V326N^; **(C)** *S*. *cerevisiae* cells expressing Als5^WT^; **(D)** *C*. *albicans* SC5314.
(TIFF)

S2 Fig Effects of vortex-mixing on ThS cell surface fluorescence. FACS analyses of populations of cells stained with ThS 1 μM. Yellow represents unstimulated cells, and blue represents vortex-mixed cells. **(A)** *S*. *cerevisiae* cells with EV; **(B)** *S*. *cerevisiae* cells expressing Als5p^V326N^; **(C)** *S*. *cerevisiae* cells expressing Als5^WT^; **(D)** *C*. *albicans* SC5314.
(TIFF)

S3 Fig Effects of vortex-mixing on heat killed Als5p-expressing cells. Cells were vortex-mixed or not then stained with ThT (500 nM). Scale bars represent 5μm.
(TIFF)

S1 Table Effect of vortex-mixing on mean ThS fluorescence of yeast cells. (PDF)
