# Supplementary figures and images for: Quantitative Analyses of Force-Induced Amyloid Formation in Candida albicans Als5p: Activation by Standard Laboratory Procedures
Source: PLoS One. 2015 Jun 5;10(6):e0129152. doi: 10.1371/journal.pone.0129152 (PMC4457901; doi:10.1371/journal.pone.0129152)

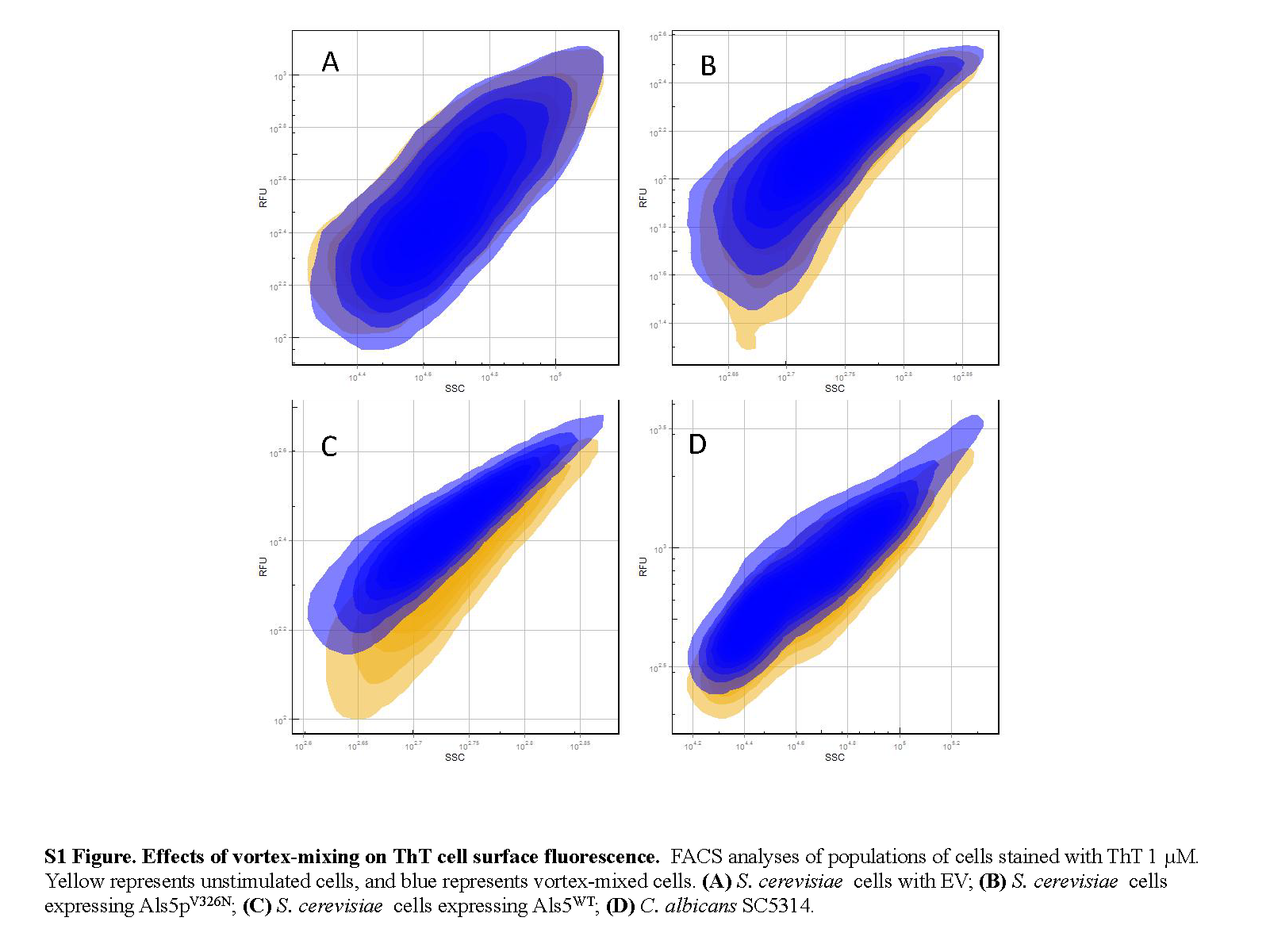

Supplement: S1 Fig — FACS analyses of populations of cells stained with ThT 1 μM. Yellow represents unstimulated cells, and blue represents vortex-mixed cells. (A) S. cerevisiae cells with EV; (B) S. cerevisiae cells expressing Als5pV326N; (C) S. cerevisiae cells expressing Als5WT; (D) C. albicans SC5314. (TIFF) [file pone.0129152.s001.tiff]

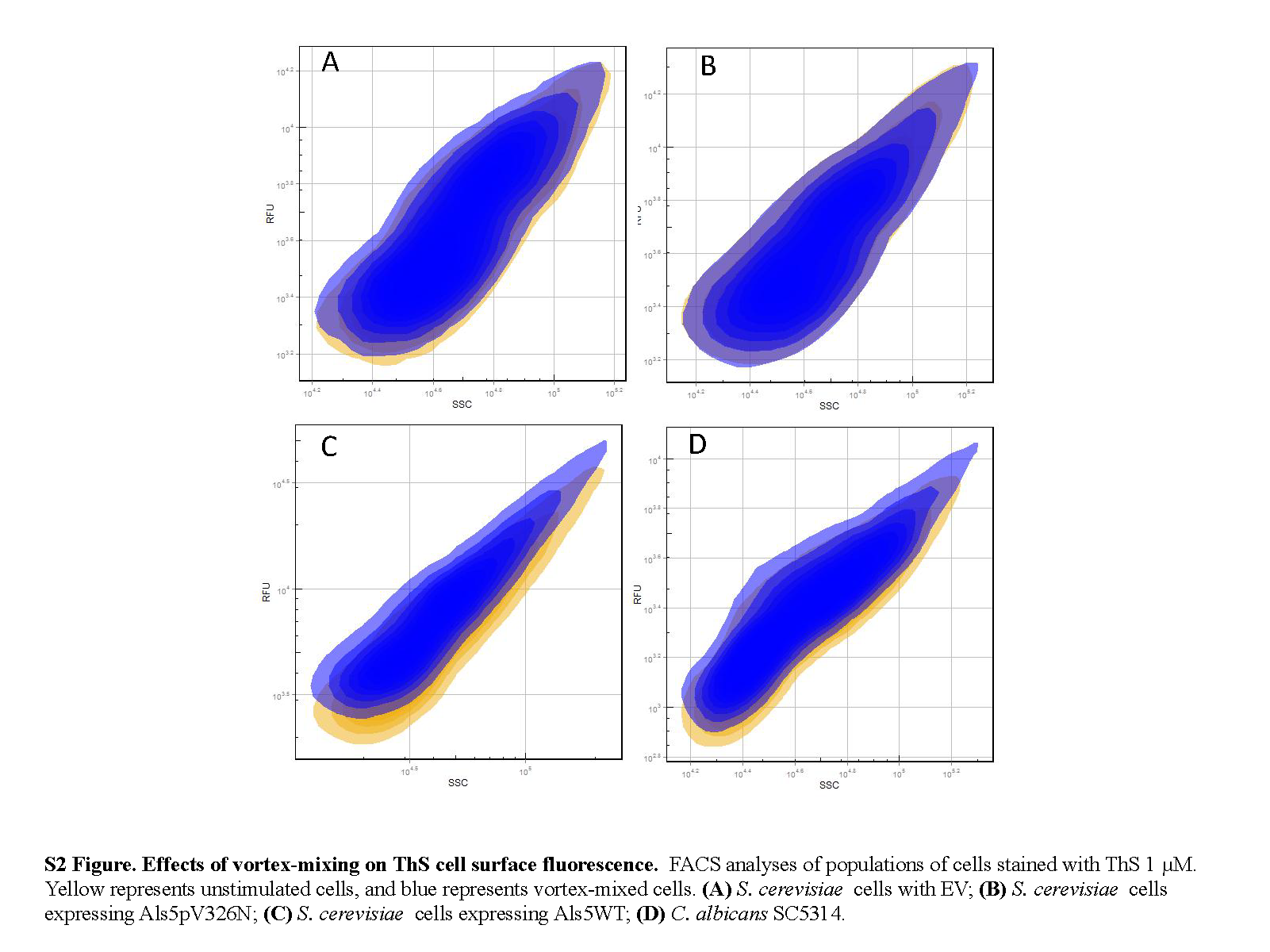

Supplement: S2 Fig — FACS analyses of populations of cells stained with ThS 1 μM. Yellow represents unstimulated cells, and blue represents vortex-mixed cells. (A) S. cerevisiae cells with EV; (B) S. cerevisiae cells expressing Als5pV326N; (C) S. cerevisiae cells expressing Als5WT; (D) C. albicans SC5314. (TIFF) [file pone.0129152.s002.tiff]

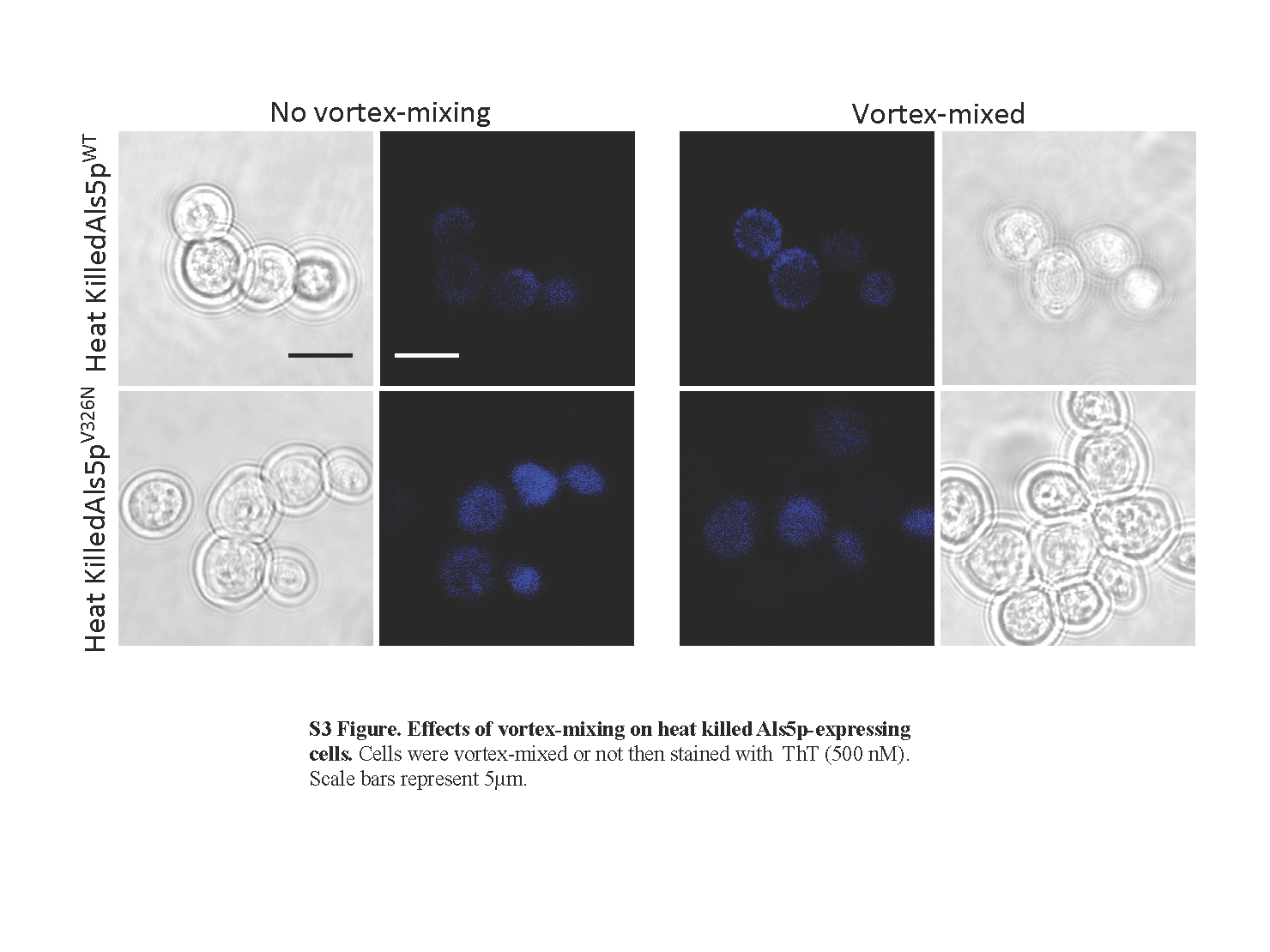

Supplement: S3 Fig — Cells were vortex-mixed or not then stained with ThT (500 nM). Scale bars represent 5μm. (TIFF) [file pone.0129152.s003.tiff]
