# Supplementary material for: Quantitative Analyses of Force-Induced Amyloid Formation in Candida albicans Als5p: Activation by Standard Laboratory Procedures
Source: PLoS One. 2015 Jun 5;10(6):e0129152. doi: 10.1371/journal.pone.0129152 (PMC4457901; doi:10.1371/journal.pone.0129152)
Supplement: S1 Table — (PDF) [file pone.0129152.s004.pdf]

Supporting Information

S1 Table. Effect of vortex-mixing on mean ThS fluorescence of yeast cells.

Cells	Vortex-mixed	Fluorescence (mean±se)*	Ratio
C. albicans SC5314	-	2315 ±93	1.3± .133
	+	2890±365	
S. cerevisiae (Als5)	-	6344±391	1.3± .103
	+	7965±656	
S. cerevisiae (V326N)	-	4628±171	1.03±0.072
	+	4761±301	
S. cerevisiae (EV)	-	4442±308	1.01±0.073
	+	4478±100	

*Geometric mean and s.e. for 3 determinations
